# Autologous Mesenchymal Stem Cells for Treatment of Chronic Active Antibody-Mediated Kidney Graft Rejection: Report of the Phase I/II Clinical Trial Case Series

**DOI:** 10.3389/ti.2022.10772

**Published:** 2022-11-22

**Authors:** Željka Večerić-Haler, Matjaž Sever, Nika Kojc, Philip F. Halloran, Emanuela Boštjančič, Gregor Mlinšek, Manca Oblak, Primož Poženel, Urban Švajger, Katrina Hartman, Miomir Kneževič, Ariana Barlič, Lenart Girandon, Andreja Aleš Rigler, Samo Zver, Jadranka Buturović Ponikvar, Miha Arnol

**Affiliations:** ^1^ Department of Nephrology, University Medical Centre Ljubljana, Ljubljana, Slovenia; ^2^ Faculty of Medicine, University of Ljubljana, Ljubljana, Slovenia; ^3^ Department of Haematology, University Medical Centre Ljubljana, Ljubljana, Slovenia; ^4^ Institute of Pathology, Faculty of Medicine, University of Ljubljana, Ljubljana, Slovenia; ^5^ Division of Nephrology and Transplant Immunology, Alberta Transplant Applied Genomics Centre, University of Alberta, Edmonton, AB, Canada; ^6^ Division for Cells and Tissue, Blood Transfusion Centre of Slovenia, Ljubljana, Slovenia; ^7^ Educell d.o.o Cell Therapy Service, Ljubljana, Slovenia

**Keywords:** kidney transplant, mesenchymal stem cells, stem cells, antibody mediated rejection, kidney allograft

## Abstract

Mesenchymal stem cell (MSCs) therapy has already been studied in kidney transplant recipients (KTRs), and the available data showed that it is safe and well tolerated. The aim of this study was to evaluate the safety and efficacy of autologous MSCs in combination with standard therapy in KTRs with biopsy-proven chronic active antibody-mediated rejection (AMR). Patients with biopsy-proven chronic active AMR received treatment with autologous bone marrow-derived MSCs (3 × 10^6^ cells/kg iv) after completion of standard therapy and were followed for up to 12 months. The primary endpoints were safety by assessment of adverse events. Secondary endpoints included assessment of kidney graft function, immunological and histological changes related to AMR activity and chronicity assessed by conventional microscopy and molecular transcripts. A total of 3 patients were enrolled in the study before it was terminated prematurely because of adverse events. We found that AMR did not improve in any of the patients after treatment with MSCs. In addition, serious adverse events were observed in one case when autologous MSCs therapy was administered in the late phase after kidney transplantation, which requires further elucidation.

## Graphical Abstract

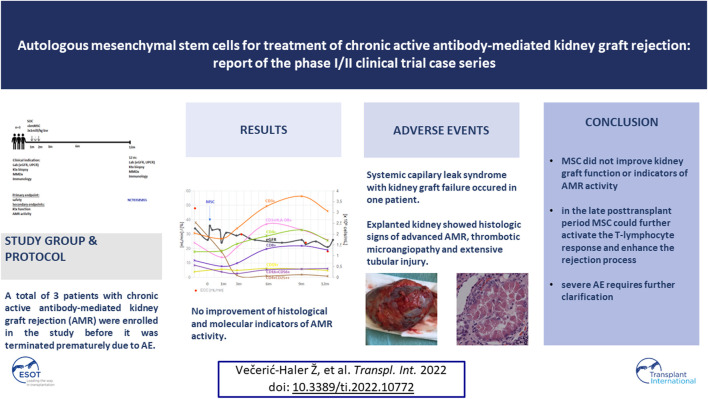

## Introduction

Chronic antibody-mediated rejection (AMR) is a major challenge to long-term graft survival in kidney transplant recipients (KTRs) (1). New technologies, including genomic studies to improve the specificity and sensitivity of renal biopsies such as Molecular Microscope Diagnostic System (MMDx) (2) and assays to detect donor-specific antibodies (DSAs), have provided important insights into the pathophysiology and diagnosis of chronic AMR. Unfortunately, these advances have not yet translated into improved outcomes because, in the absence of therapies that can suppress the formation of antibodies by plasma cells, available therapies can only slow the progression of graft injury.

Mesenchymal stem cells (MSCs) have attracted much interest due to their immunomodulatory properties (3). In kidney transplantation, MSCs have been used in a number of small and two large studies to induce immune tolerance, treat and prevent T-cell rejection, reduce interstitial fibrosis/tubular atrophy, minimize nephrotoxic immunosuppressants (4–13), and more recently to target chronic AMR resistant to conventional therapies (Supplementary Table S1) (14–16)**.** With the exception of few studies using third-party MSCs (4, 11, 14–16), autologous or kidney donor-derived cells were used to avoid alloimmunization. In the recent pilot study by (14) who were the first to report the use of allogeneic bone marrow-derived MSCs (bmMSCs) in two KTRs with chronic active AMR refractory to rituximab and intravenous immunoglobulin, no improvement in graft function was observed. In contrast (15), recently demonstrated the efficacy of allogeneic bmMSCs in 23 KTRs in improving graft function and survival compared with matched controls after 2 years of follow-up. No association between MSC therapy and serious complications was observed in these studies (17, 18).

The therapeutic mechanism of MSCs has not been fully elucidated. With regard to organ transplantation, MSCs have been shown to induce long-term graft acceptance by *in vivo* generation of regulatory T cells and suppression of T cell proliferation in response to autoantigens and alloantigens in a non-MHC-linked manner (19). In the context of humoral response, preclinical studies have shown that MSCs can reduce circulating allospecific antibodies and allospecific IgG deposition in the graft, with these effects being mediated by regulatory T cells (20–22).

Here, we report the safety and efficacy of a 12-month follow-up of a case series of patients with chronic active AMR who received autologous bmMSCs in combination with standard of care (SOC) therapy at a late stage after kidney transplantation. Patients were enrolled in the study protocol (ClinicalTrials.gov, number NCT03585855), which was discontinued due to serious adverse events, including kidney graft loss in one patient (published elsewhere) (23).

## Materials and Methods

### Study Design

This was a prospective, investigator-initiated, interventional, single-center clinical study. The study was approved by the National Medical Ethics Committee of the Republic of Slovenia (permit number 0120-215/2018-4) and conducted in accordance with the Declaration of Helsinki. Written informed consent was obtained from all participants. The inclusion and exclusion criteria are presented in the Supplementary Material. The trial is registered with ClinicalTrials.gov, NCT03585855.

### Procedures

**FIGURE 1:**
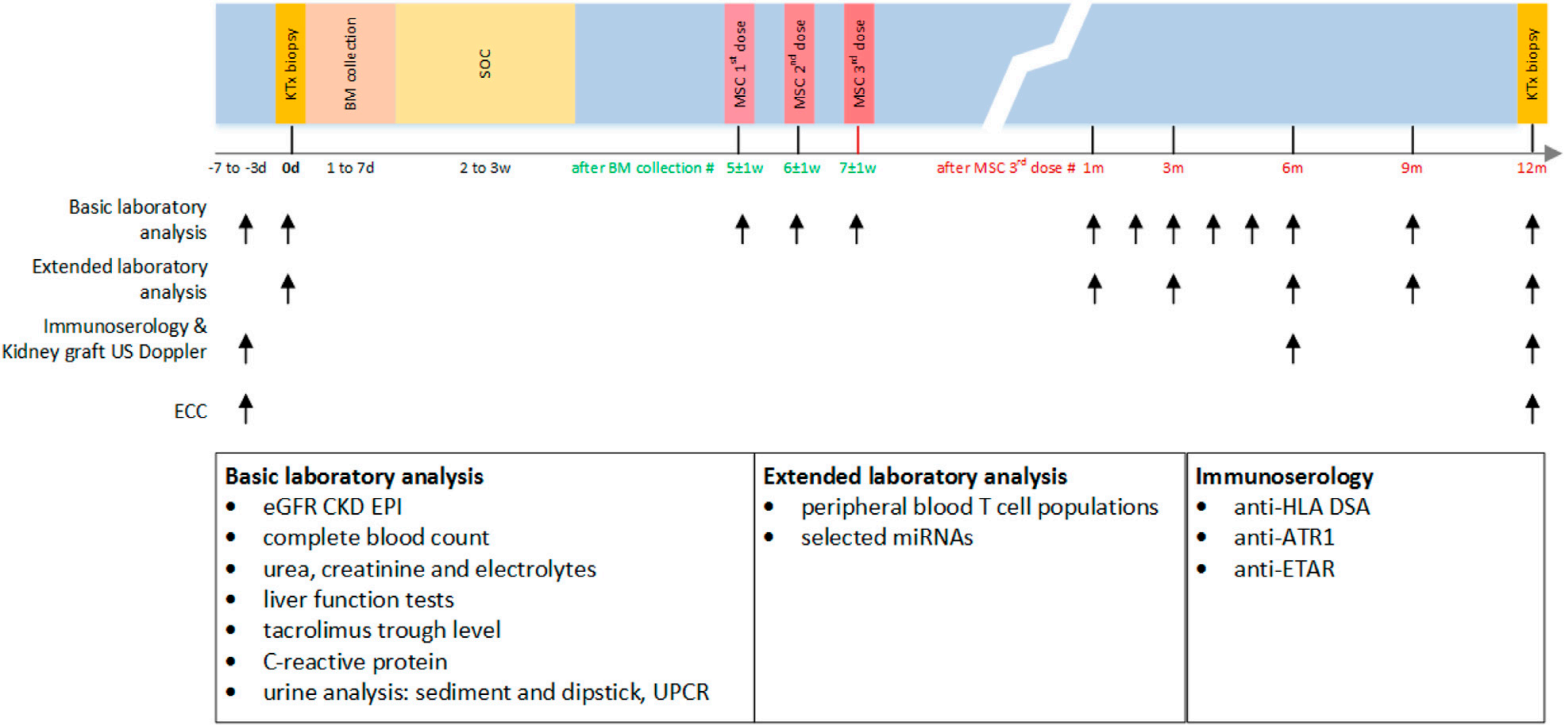
Study design. BM, bone marrow; w, week; d, day; KTx, kidney transplant; SOC, standard of care treatment (including plasmapheresis 7x, intravenous immunoglobulins 100 mg/kg 7x, and corticosteroids); MSC, mesenchymal stem cell therapy; eGFR CKD EPI, estimated glomerular filtration rate according to the Chronic Kidney Disease Epidemiology Collaboration formula; ECC, excreted creatinine clearance; US, ultrasound; UPCR, urinary protein:creatinine ratio; HLA, human leukocyte antigen; DSA, donor specific antibodies; ATR1, antibodies against angiotensin receptor type 1; ETAR, antibodies against endothelin-1 type A receptor; miRNA, microRNA expression.

The study design is shown in Figure 1. All participants received SOC immunosuppression for chronic active AMR (including plasmapheresis 7x, intravenous immunoglobulins (IVIg) 100 mg/kg 7x, and corticosteroids) followed by 3 infusions of autologous bmMSCs at a single dose of 1 × 10^6^ cells/kg 1 week apart (total 3 × 10^6^ cells/kg). The protocol was developed based on data from studies in experimental animal models, clinical data on previously experimental MSC treatment of renal pathologies, and treatment results of graft-versus-host disease in allogeneic stem cell transplant setting. Our center’s extensive experience with various experimental stem cell treatments also influenced the development of study design. During the follow-up period of up to 12 months, patients were monitored for adverse events according to CTCAE 5.0; estimated glomerular filtration rate (eGFR) was determined according to the Chronic Kidney Disease Epidemiology Collaboration formula (CKD-EPI) monthly for the first 6 months and once every 3 months thereafter; excreted creatinine clearance (ECC) before and after 12 months; kidney transplant Doppler ultrasound at 0, 6, and 12 months; anti-HLA DSAs, antibodies to angiotensin receptor type 1 (anti-ATR1), and anti-endothelin 1 type A receptor antibodies (anti-ETAR) at 0, 6, and 12 months; immunophenotyping of peripheral blood T-cell populations and selected miRNA expression at 0, 1, 3, 6, 9, and 12 months; kidney biopsies including analysis of molecular transcripts by MMDx before and 12 months after MSCs application.

### Mesenchymal Stem Cell Preparation and Culture Protocol

MSCs were prepared and cultured as described in the Supplementary Material, where data on viability and phenotypic characteristics of MSC therapy are also listed.

### Kidney Graft Function

Kidney function was assessed by eGFR, calculated using the CKD-EPI study formula with serum creatinine (s-Cr), and by 24-hour urine collection and measurement of ECC.

### Conventional and Molecular Kidney Graft Biopsy Assessment

Scoring of kidney biopsies and histological diagnosis of AMR were performed in a blinded fashion by a renal pathologist, using the 2019 Banff classification (24). Immunohistochemical staining, including CD44 (dilution 1:200; Cell Marque, Rocklin, United States) and CD105 (dilution 1:100; Epitomics, Burlingame, United States) was performed on kidney graft biopsies 12 months after MSCs application. Precision molecular assessment of kidney transplant biopsies was performed with MMDx using the reported protocol (24).

### RNA Isolation and miRNA Quantification

Expression of selected miRNAs was determined by Quantitative real-time polymerase chain reaction (qPCR). The details are provided in the Supplementary Material.

### Immunological Monitoring

Details of HLA, anti-ETAR, and anti-ATR1 antibodies, peripheral blood lymphocyte populations, and serum cytokine analyses are provided in the Supplementary Material.

### Outcomes

Primary outcome measures were safety of MSC therapy assessed by adverse events at 12 months. Secondary outcome measures included changes in kidney graft function, histology, MMDx scores, and miRNA expression during the 12-month follow-up.

### Statistical Analysis

All the analyses in the study were descriptive and all graphs were created using Microsoft Excel 2021.

## Results

**FIGURE 2:**
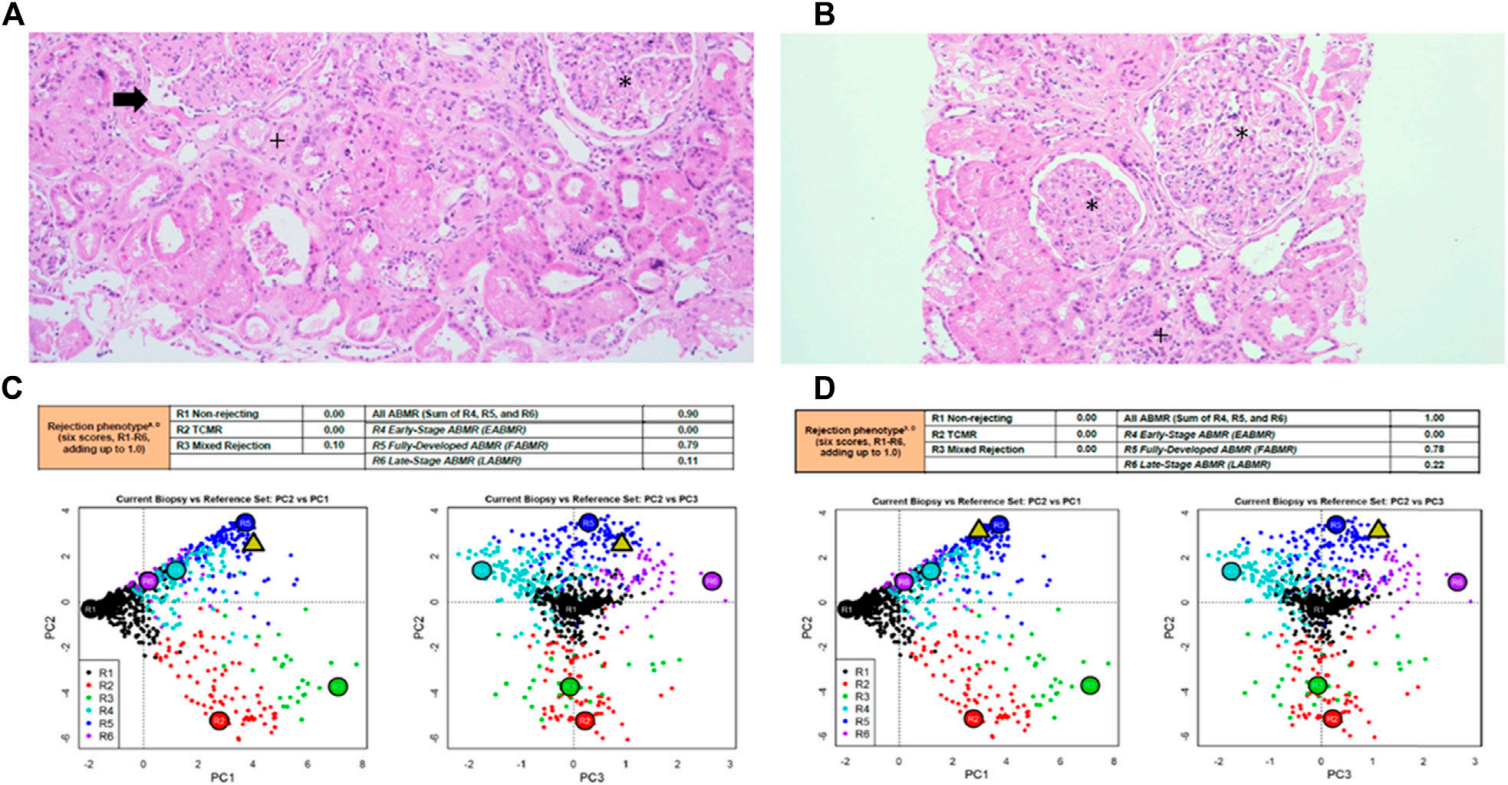
Kidney graft histopathology and molecular microscope diagnostic report reports in patient #1 and patient #2. Kidney graft histopathology 12 months after application of MSCs in patient #1 **(A)** and patient #2 **(B)**, hematoxylin eosin, x100. Molecular microscope diagnostic report (MMDx) for kidney transplant biopsy in patient #1 **(C)** and in patient #2 **(D)** 12 months after application of MSCs showing fully-developed antibody-mediated rejection with severe microvascular inflammation and molecular features of extensive interstitial fibrosis/tubular atrophy. *glomerulitis with transplant glomerulopathy (double contour formation), **+**interstitial fibrosis and tubular atrophy, **→** peritubular capillaritis.

**FIGURE 3:**
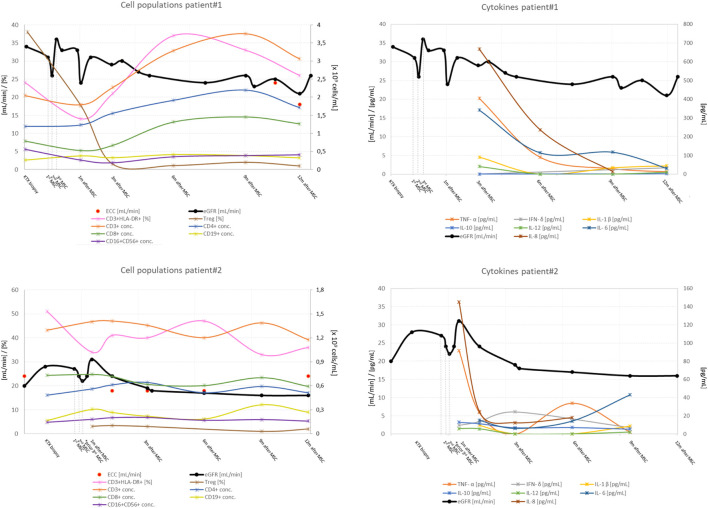
Kidney graft function, peripheral blood lymphocyte populations and serum concentration of cytokines before and after application of MSCs in patient #1 and patient #2. Legend: eGFR, estimated glomerular filtration rate; conc, concentration; Treg, CD4^+^CD25^++^ T cells (this subset contains a proportion of Tregs cells). Cytokine concentrations after mesenchymal stem cells application (in U/ml for soluble interleukin 2 receptor and in pg/ml for other cytokines); TNF, tumor necrosis factor; IL, interleukin; s-IL-2-R, soluble interleukin 2 receptor; IFN, interferon, *patient#2 received only two doses of MSCs.

Baseline and end of follow-up characteristics of patients are presented in Table 1. Kidney graft histopathology and MMDx reports are shown in Table 1 and Figure 2, kidney graft function, peripheral blood lymphocyte populations and serum concentration of cytokines are presented in Figure 3, with additional details provided in the Supplementary Material.

**TABLE 1:** Baseline and end of follow-up characteristics of patients treated with MSCs.

	Patient #1			Patient #2			Patient #3	
Cause of end stage kidney disease	Autosomal dominant polycystic kidney disease			Reflux nephropaty			IgA nephropathy	
Time after Tx (years)	9			9			5	
Age	53			56			26	
Sex	Male			Male			Male	
Maintenance IS	cyclosporine, MMF			cyclosporine, MMF			tacrolimus, MMF, steroid	
**Basic kidney graft function and proteinuria prior to and 12 months after MSCs**								
s-Cr (μmol/L)	189	240		246	347		240	NA-dialysis dependant
ECC (ml/min)	48	18		24	18		24	NA
eGFR (ml/min/1.73m^2^)	34	21		20	17		28	NA
Proteinuria (g/day)	0.75	1.5		1.3	1.75		3.4	NA
**Immune monitoring prior to, 6 and 12 months after MSCs**								
HLA DSA specifity (MFI) prior, 6, and 12 months after MSCs	DQA1 (530)	DQA1 (140)	DQA1 (190)	DQA1 (1940) DQB1 (460)	DQA1 (1830), DQB1 (240)	DQA1 (1390), DQB1 (210)	DQB1 (3390), DQA1 (2520)	NA
ATR1 (U/ml) antibodies prior to, 6, and 12 months after MSCs	5.5	4.6	6.3	45.6 (positive)	59.8 (positive)	63.2 (positive)	5.9	NA
ETAR (U/ml) antibodies prior to, 6, and 12 months after MSCs	8.6	3.3	6.0	48.2 (positive)	45.0 (positive)	57.8 (positive)	4.9	NA
**Banff score in renal tansplant biopsies prior to and 12 months after MSCs administratin**								
Bannf score	t0,i1, ti1, v0, ptc2 cv2, g2, cg3, mm1, ci1, ct1, ah2, i-IFTA2, C4d0, t-IFTA0, ptcml1, pvl0	t0,i1, ti2, ptc2, v0, cv2, g2, cg3, mm1, ci2, ct2, ah2, i-IFTA2, C4d0, t-IFTA0, ptcml2, pvl0		t0, i1, ti2, v0, ptc3 cv2, g3, cg3, mm1, ci1, ct1, ah3, i-IFTA1, C4d0, t-IFTA0, ptcml3, pvl0	t0, i1, ti2, v0, ptc3 cv2, g2, cg3, mm1, ci2, ct2, ah3, i-IFTA2, C4d0, t-IFTA0, ptcml3, pvl0		t0, i2, ti2, v1, ptc3 cv2, g3, cg3, mm1, ci2, ct2, ah2, i-IFTA3, C4d0, t-IFTA1, ptcml3, pvl0	t3, i3, ti3, v3, ptc3 cv3, g3, cg3, mm3, ci3, ct3, ah2, i-IFTA3, C4d0, t-IFTA2, ptcml3, pvl0, thrombotic microangiopathy, severe tubular damage

Tx-transplantation; IS-immunosuppression; MMF-mycophenolate mofetil; s-Cr-serum creatinine; ECC-excreted creatinine clearence; eGFR-estimated glomerular filtration rate; HLA-human leukocyte antigen; DSA-donor specific antibodies; ATR1- antibodies against angiotensin receptor type 1; ETAR-antibodies against endothelin-1 type A receptor; t-tubulitis; i-inflammation in non-scarred cortex; ti-total cortical inflammation; v- endarteritis; ptc-peritubular capillaritis; cv-arterial intimal fibrosi;, g-glomerulitis; cg-transplant glomerulopathy; mm-mesangial matrix expansion; ci-interstitial fibrosis in cortex; ct;tubular atrophy, ah-artriolar hyalinosis; i-IFTA inflammation in scarred cortex; C4d; linear staining in ptc or medullary vasa recta by immunofluorescence, t-IFTA- tubulitis in tubules within scarred cortex; ptcml-peritubulr capillary basement membrane multilayering; pvl- intrarenal polyomavirus load level. For details regarding Banff scoring schemes, see Loupy et al, Am J Transplant, 2020;20:2318-2331.

### Patient #1

Patient #1 was a 53-year-old man with end stage kidney disease due to ADPKD who received the first deceased donor kidney transplant 9 years before enrollment in the study. After histological diagnosis of chronic active AMR, patient received SOC therapy, followed by MSCs 3 × 10^6^/kg at 1-week intervals. He reported no adverse events. Histologic assesment before therapy revealed focal glomerulitis and 30% moderate peritubular capillaritis without tubulitis, double contour formation (transplant glomerulopathy) in 10 of 21 glomeruli, 20% interstitial fibrosis/tubular atrophy, and a lymphocytic interstitial infiltrate in areas of interstitial fibrosis (i-IFTA2). A very sparse lymphocytic interstitial infiltrate consisting of CD3+T lymphocytes, scarce CD68^+^ macrophages, and few CD79a+B lymphocytes was present in another 20% of the preserved renal cortex. Immunofluorescence for C4d in the peritubular capillaries was negative. Electron microscopy showed peritubular capillary basement membrane multilayering (ptcml1), see Table 1.

During follow-up, patient experienced a continuous decrease in kidney function and an increase in proteinuria. The MFI values of DSAs decreased after standard and MSC therapy and remained stable thereafter. Surveillance kidney biopsy at 12 months showed similar histologic features to the previous biopsy, with a decrease in peritubular capilaritis (from 30% to 15%) and an increase in chronicity (30% interstitial fibrosis/tubular atrophy and ptcml2). The number of CD3^+^ lymphocytes, CD79a+ lymphocytes, and CD68^+^ macrophages was similar to the biopsy before MSCs administration. MMDx analysis 12 months after MSCs therapy showed persistence of fully developed AMR with molecular classifiers of inflammation and fibrosis in the range of highly elevated values.

After MSCs infusion, the concentration of helper CD3^+^CD4^+^ and cytotoxic CD3^+^CD8^+^ T lymphocytes increased. The proportion of activated CD3^+^HLA-DR^+^ lymphocytes increased, whereas the absolute number and proportion of CD4^+^CD25^++^ T lymphocytes within the total CD4^+^ population decreased. CD4^+^CD25^++^ T lymphocytes were still suppressed 12 months after MSCs infusion. NK (CD16^+^CD56^+^) and B lymphocyte (CD19^+^) concentrations were consistent with patient age and stable throughout the follow-up period**.** MSC therapy had no effect on memory T cells. Evaluation of timely expression of miRNA associated with AMR showed no significant changes in expression profiles during the observation period (Supplementary Figure S1).

### Patient #2

Patient #2 was a 56-year-old man with end stage kidney disease due to reflux nephropathy. He received his second deceased donor kidney 9 years before participation in the study and had mixed acute AMR and T-cell rejection 5 years after transplantation, which was treated with steroid pulses, plasmapheresis, IVIg, and additional therapy with rituximab and bortezomib. Kidney graft function remained stable for 4 years but steadily deteriorated in the last 2 months before enrollment.

After graft biopsy that revealed chronic active AMR with anti-HLA DSAs, anti-ATR1 and anti-ETAR antibodies, he received SOC treatment, followed by 2 × 10^6^/kg MSCs at 1-week intervals. After the second dose, we observed an increase in s-Cr and noninfectious diarrhea, and because of the observed side effects, the third dose of MSCs was withheld. The potential side effects resolved spontaneously.

During follow-up, kidney function decreased and proteinuria increased. Although the concentration of T lymphocytes remained unchanged, the proportion of activated T lymphocytes increased, while at the same time the proportion and absolute number of CD4^+^CD25^++^ T lymphocytes continued to decrease after treatment with MSCs. The concentration of memory T lymphocytes was not affected by treatment. Surveillance kidney biopsy 12 months after MSC treatment revealed persistent chronic active AMR, with anti-HLA DSAs, anti-ETAR, and anti-ATR1 antibodies still present. Histologically, there was global glomerulitis with transplant glomerulopathy, diffuse peritubular capillaritis, and an increase in chronic Banff scores: intersitital fibrosis (from 15% to 35%), tubular atrophy (from 10% to 30%), and peritubular capillary basement membrane multilayering (from ptcml 1 to ptcml3), see Table 1. The amount of CD3^+^ lymphocytes, CD79a+ lymphocytes, and CD68^+^ macrophages were similar to those obtained by biopsy before MSCs were administered. MMDx 12 months after MSC treatment showed molecular classifiers indicative of microvascular inflammation and fibrosis associated with persistent chronic active AMR. Expression of miRNA associated with AMR also showed no significant changes in expression profiles during the observation period (Supplementary Figure S1).

### Patient #3

Patient #3 was a 26-year-old man with end stage kidney disease due to IgA nephropathy. He received a deceased kidney transplant 4 years before participation in the study. Two years after transplantation, he was diagnosed with mixed T-cell rejection (Banff 4/IB) and AMR treated with high-dose steroids, plasmapheresis, antithymocyte globulin, and rituximab. After 3 years of stable kidney function, s-Cr and proteinuria increased in the last months before enrollment. Graft biopsy revealed chronic active AMR. Because he had a history of childhood acute lymphoblastic leukemia, we performed a bone marrow aspiration before entering the study, which showed mild reactive changes. After completion of SOC therapy, he received MSCs (3 × 10^6^ cells/kg) according to the study protocol.

Severe systemic side effects occurred after the third administration of MSCs, including acute noninfectious gastroenteritis, ascites, splenomegaly, resistant hypertension, hemolytic anemia, pancytopenia, and nephrotic range proteinuria. After the third administration of MSCs, his kidney function deteriorated (s-Cr 390 μmol/L, eGFR 10 ml/min/1.73 m^2^) and kidney graft explantation had to be performed 2 months after the MSCs administration. The full course and temporal evolution of the adverse reaction including histopathological changes have been described in detail previously (23).

## Discussion

Here we present the results of the first phase I/II case series of KTRs with chronic active AMR treated with autologous bmMSCs in combination with SOC treatment in the late period after kidney transplantation.

In our centre, the standard treatment protocol for chronic active AMR consists of corticosteroids, plasmapheresis, and IVIg. In patients who do not respond to the SOC therapy, rituximab and bortezomib have been used in the past. However, this did not improve graft function and survival, while the risk of such potentiated therapies for life-threatening side effects increased (25, 26). Because of the disappointing treatment results, we developed a study protocol to investigate the safety and efficacy of therapy with autologous bmMSCs superimposed on standard therapy. Autologous MSCs were chosen instead of third-party MSCs to prevent alloimmunization. Unfortunately, due to premature study termination, only three patients could be enrolled, two of whom are presented in detail here (patient #1 and #2), while the course of patient #3, who experienced serious adverse events in the form of systemic capillary leak syndrome requiring discontinuation of the study protocol, has been described elsewhere (23).

The clinical trials of MSCs in kidney transplantation published through December 2021 (Supplementary Table S1) are mainly phase I or early phase II studies in which MSCs were administered before, at or early after transplantation against a background of regular immunosuppression to induce immunologic tolerance. With the exception of four reported studies (4, 11, 14, 15), the MSCs used were of autologous origin. Only two studies reported the administration of MSCs in the late period after kidney transplantation (14, 15). These studies have shown that treatment with MSCs is safe and feasible.

While patient #1 experienced no adverse events after MSCs administration, patient #2 experienced worsening graft function and grade 1 diarrhea immediately after the second administration of MSCs. Because patient #3 had already experienced serious adverse events during this period, which also started with noninfectious diarrhea after the second MSCs administration and increased to fully developed systemic capillary leak syndrome, we decided not to continue the third MSCs administration in patient #2. Diarrhea gradually resolved, and graft function stabilized. Noninfectious diarrhea in a KTR who had received allogeneic MSCs was recently described in a study by (14).

There appears to have been a transient MSC-mediated impairment of graft function in the period up to 1 month after MSC infusion, which returned to baseline in patient #1 and patient #2. This observation may be related to the timing of MSCs infusion in relation to the timing of transplantation. When used after kidney transplantation, transient graft dysfunction occurred, which was not observed when the infusion was applied before transplantation (7, 8). This observation is consistent with previous experimental models in which rodents developed kidney dysfunction, presumably as a consequence of preferential homing of the infused cells at the site of tissue injury, which releases chemotactic signals such as hyaluronic acid (27) or complement components (28). In the absence of chemotactic signals, such as during stem cell infusion before allografting or when experimentally antagonized by complement inhibitors, MSCs preferentially recruit to lymphoid organs without graft dysfunction, increasing numbers of T regulatory cells (Tregs) and inducing long-term graft acceptance (29).

After returning to baseline, the function of the transplanted kidney slowly deteriorated over a 12-month period in patient #1 and patient#2. End-stage kidney graft failure occurred 3 and 2 years after AMR treatment, respectively, which is consistent with treatment outcomes in our historical cohorts of patients with chronic active AMR, in whom the 1-year survival rate of a transplanted kidney was 56% and the 3-year survival rate was 41% (25, 26, 30). Anti-HLA DSAs were present in all three patients before treatment, and their MFI levels decreased after treatment with standard therapy in combination with MSCs. Histopathological findings before and 12 months after MSC treatment in patient #1 and patient #2 showed comparable chronic changes in all parts of the nephron. No CD105^+^CD44^+^ (markers co-expressed by MSCs) or ectopic tissue infiltrates, which would indicate transdifferentiation of infused MSCs, were found in the biopsy specimens. Molecular analysis of the kidney biopsy before and after treatment showed that the classifiers of fully developed severe AMR, including g-, cg-, and ptc-related molecular features, persisted. Previously, we identified miRNAs (*miR-29c, miR-126, miR-146a, miR-150, miR-155, and miR-223*) which are typically expressed in patients with AMR (31). Selected miRNAs analysis in MSC-treated patients after MSCs application did not show any significant visible changes in their expression.

After MSC therapy, the percentage of activated T lymphocytes increased. Analysis of T-cell differentiation showed an increased Th1/Th2 ratio with decreasing numbers and ratios of CD4^+^CD25^++^ T lymphocytes (i.e., CD4^+^CD25^high^ cells that express a high level of CD25 and may contain a proportion of Tregs) during the observation period. MSC therapy had no effect on the number of NK cells and B lymphocytes. Despite an increased percentage of activated T lymphocytes in the peripheral blood, we observed no increase in interstitial inflammation, peritubular capillaritis, or other signs of activity in the renal transplant biopsies compared with the biopsies before MSCs were administered in patient#1 and #2. However, in patient# 3, severe glomerular and tubular damage with endarteritis and thrombotic microangiopathy were noted, as we reported previously. The results suggest that MSC therapy does not alleviate rejection by enhancing the regulatory immune cell component. Rather, it may be responsible for a transient activation of the T-lymphocyte response, which in some cases may enhance the rejection process. The results of our immune monitoring do not coincide with the general knowledge regarding MSCs function both *in vivo* and *in vitro*. For example, Carrion et al (32) and Casiraghi et al (28) showed that MSCs suppress the proliferation, activation, and differentiation of Th1 and Th17 cells and increase the proportion of regulatory T cells when added at the beginning of the polarization process. In addition, MSCs can also suppress proliferation and activation of differentiated Th1 and Th17 cells. Such conflicting results are difficult to interpret and could be related to the quality of MSC products. On the other hand, they could reflect the functional plasticity of MSCs in a specific clinical setting. For MSCs to fully develop their immunosuppressive potential *in vivo*, they first need to undergo proper licensing by the inflammatory environment (33). In this manner, MSCs therapy was successful in a graft-versus-host disease setting with an extensive inflammatory microenvironment (34), whereas its use was detrimental in a heart transplant model where recipients were pretretated with MSCs in the absence of inflammatory stimuli (35). Furthermore, certain microenvironment factors (such as toll-like receptor ligands) have been shown to induce a pro-inflammatory MSC type, that can support T cell activation (36).

Although we currently have limited data related to the results of AMR treatment with MSCs, the largest research to date^15^ has shown that allogeneic MSCs in combination with immunosuppressive drugs are effective in terms of delaying the deterioration of graft function, probably by decreasing anti-HLA DSAs levels and reducing DSA-induced injury. Unfortunately, our case series results could not confirm this. This discrepancy may be due to the use of autologous MSCs with potentially poor quality and immunomodulatory efficacy of bmMSCs obtained from patients with advanced graft failure and long-term treatment with bone marrow immunosuppressants. Prior exposure of bone marrow to chemotherapeutic agents may lead to alterations in the expansion capacity, phenotype, and DNA injury of MSCs, resulting in genetic instability and therapy-related malignancy (37, 38). MSCs obtained from patients with advanced kidney failure have been shown to be of lower quality (39). Similarly, MSCs in our cases exhibited altered morphology with more flattened cells than would have been expected for early culture **(Supplementary Material)**. The impact of above-mentioned factors on outcome in our patients is difficult to assess, but given the data from preclinical studies in similar cases (i.e., uremic milieu, distorted stem cell niche, use of immunosuppressants), decreased MSC function might be expected.

## Conclusion

Taken together, the administration of autologous MSCs in the three patients with chronic active AMR did not improve kidney graft function and had no protective effect on histological and molecular indicators of AMR activity. From an immunological perspective, treatment with autologous MSCs, when given in the late posttransplant period, could further activate the T-lymphocyte response, which may enhance the rejection process. The safety of MSC treatment in patients after solid organ transplantation should be closely monitored for the occurrence of as-yet unexplained adverse reactions. Further studies with prolonged follow-up are needed before continuing MSCs administration to patients in the late period after transplantation.

## Study Limitations

The main limitation of the study is the small sample size, as the study was terminated prematurely due to serious adverse events in one of the patients. As a result, the originally planned comparison cohort of patients treated with SOC alone was not included. Another shortcoming that may have affected the treatment outcome is that we used a slightly modified protocol for bone marrow isolation and MSCs preparation in patient#2 to ensure a less invasive bone marrow collection.

## Data Availability

The original contributions presented in the study are included in the article/Supplementary Material, further inquiries can be directed to the corresponding author.
