# Supplementary material for: Autologous Mesenchymal Stem Cells for Treatment of Chronic Active Antibody-Mediated Kidney Graft Rejection: Report of the Phase I/II Clinical Trial Case Series
Source: Transpl Int. 2022 Nov 22;35:10772. doi: 10.3389/ti.2022.10772 (PMC9722440; doi:10.3389/ti.2022.10772)
Supplement: Supplementary file 1 [file DataSheet1.docx]

**Supplementary material**

**Supplementary Table 1. Registered clinical studies with MSCs used in kidney transplant recipients up to December 2021.** Legend: KTR-kidney transplant recipient, cAMR-chronic antibody mediated rejection, MSC-mesenchymal stem cells.

| **Reference** | **Number of participants** | **Dose and type of stem cells/time of appliaction** | **Design and outcome** |
| --- | --- | --- | --- |
| 1. Perico N, Casiraghi F, Introna M, et al. Autologous mesenchymal stromal cells and kidney transplantation: a pilot study of safety and clinical feasibility. Clin J Am Soc Nephrol. 2011;6(2):412-422. | 2 KTRs of living-related kidney graft donors | **Induction:**  basiliximab (20 mg iv pretransplant and on day 4 posttransplant) and thymoglobulin, 0.5 mg/kg, daily from day 0 to day 6 posttransplant.  **Maintenance:**  cyclosporine A (CsA, target trough blood levels of 300 to 400 ng/ml up to day 7 postsurgery, and 100 to 150 ng/ml at month 5 posttransplantation), mycophenolate mofetil (plasma trough mycophenolic acid [MPA] levels of 0.5 to 1.5 μg/ml) and steroids.  **Treatment group:**  autologous bmMSC 7 days post Ktx- 1.7 × 10^6^ cells in pt1 and 2.0 × 10^6^ cells/kg bw in pt2.  **Control group:**  3 living-related kidney recipients were given the same induction therapy, but not MSCs. | **Design:**  Patients treated wth standard immunosuppressive therapy vs patients receiving autologous bmMSCs at day 7 post Ktx.  **Results:**  Cr levels increased 7 to 14 days after cell infusion in both MSC-treated patients. A graft biopsy in patient 2 excluded acute graft rejection, but showed a focal inflammatory infiltrate, mostly granulocytes. In patient 1 protocol biopsy at 1-year posttransplant showed a normal graft. Both MSC-treated patients are in good health with stable graft function. A progressive increase of the percentage of Treg and a marked inhibition of memory T cell expansion were observed posttransplant. Patient T cells showed a profound reduction of CD8+ T cell activity.  **Conclusions:**  Data show that MSC infusion in KTRs is feasible, allows enlargement of Treg in the peripheral blood, and controls memory CD8+ T cell function. |
| 1. Tan J, Wu W, Xu X, et al. Induction therapy with autologous mesenchymal stem cells in living-related kidney transplants: a randomized controlled trial. JAMA. 2012;307(11):1169-1177. | 105 KTRs of living-related kidney graft donors | **Treatment (induction) group:**  autologous bm MSC (1-2 x 10^6^/kg) at kidney reperfusion and two weeks later in two groups:  - standard-dose CNIs (n=53)  - low-dose CNIs (n=52).  **Control(induction) group:**  anti-IL-2 receptor antibody plus standard-dose CNIs (n=51).  **Maintenance:** steroids, mycophenolate mofetil (2.0 g/d for patients who weighed 80 kg or more or 1.5 g/d for those who weighed less than 80 kg.), and either cyclosporine (concentration 2 hours after dose, C_2_) of 1000 to 1200 ng/mL in the first trimester, 800 to 1000 ng/mL in the second trimester, and 600 to 800 ng/mL in the third trimester and beyond) or tacrolimus (targeting trough levels of 8 to 12 mg/kg for the first trimester, 5 to 8 mg/kg for the second, and 3 to 7 ng/mL for the third trimester and beyond) | **Design:**  This, single-site prospective, randomized study compares the risk-benefit profile of autologous MSC infusion vs anti-IL-2 receptor antibody (basiliximab) induction therapy for living-related donor kidney transplants**.**  **Results:**  Patient and graft survival at 13 to 30 months was similar in all groups. After 6 months, 7.5% in the autologous MSC plus standard-dose CNI group and 7.7% in the low-dose group compared with 21.6% controls had biopsy-confirmed acute rejection. None of the patients in either autologous MSC group had glucorticoid-resistant rejection, whereas 4 patients (7.8%) in the control group did. Renal function recovered faster among both MSC groups showing increased eGFR levels during the first month after surgery than the control group. Patients receiving standard-dose CNI had a mean difference of 6.2 mL/min per 1.73 m(2) and those in the low-dose CNI of 10.0 mL/min per 1.73 m(2). Also, during the 1-year follow-up, combined analysis of MSC-treated groups revealed significantly decreased risk of opportunistic infections than the control group.  **Conclusions:**  Among patients undergoing renal transplant, the use of autologous MSCs compared with anti-IL-2 receptor antibody induction therapy resulted in lower incidence of acute rejection, decreased risk of opportunistic infection, and better estimated renal function at 1 year. |
| 1. Perico N, Casiraghi F, Gotti E, et al. Mesenchymal stromal cells and kidney transplantation: pretransplant infusion protects from graft dysfunction while fostering immunoregulation. Transpl Int. 2013;26(9):867-878. | 2 KTRS of living-related kidney graft donors | **Induction:**  basiliximab (20 mg iv pretransplant and on day 4 posttransplant) and thymoglobulin, 0.5 mg/kg, daily from day 0 to day 6 posttransplant.  **Maintenance:**  cyclosporine A (CsA, target trough blood levels of 300 to 400 ng/ml up to day 7 postsurgery, and 100 to 150 ng/ml at month 5 posttransplantation), mycophenolate mofetil (plasma trough mycophenolic acid [MPA] levels of 0.5 to 1.5 μg/ml) and steroids.  **Treatment group:**  autologous bmMSC 2.0 × 10^6^ cells/kg body weight, 1 day before Ktx.  **Control group:**  historical kidney transplant recipients with a deceased donor (n = 6) given induction therapy with low‐dose thymoglobulin and the same maintenance immunosuppression. | **Design:**  Patients treated wth standard immunosuppressive therapy vs patients receiving autologous bmMSCs T at day 1 before Ktx.  **Results:**  In patient 3, MSC treatment was uneventful and graft function remained normal during 1 year follow-up. In patient 4, acute cellular rejection occurred 2 weeks post-transplant. Both patients had excellent graft function at the last observation. Circulating memory CD8(+) T cells and donor-specific CD8(+) T-cell cytolytic response were reduced in MSC-treated patients, not in transplant controls not given MSC. CD4(+) FoxP3(+) Treg expansion was comparable in MSC-treated patients with or without basiliximab induction.  **Conclusions:**  Thus, pre-transplant MSC no longer negatively affect kidney graft at least to the point of impairing graft function, and maintained MSC-immunomodulatory properties. Induction therapy without basiliximab does not offer any advantage on CD4(+) FoxP3(+) Treg expansion . |
| 1. Reinders MEJ, de Fijter JW, Roelofs H, et al. Autologous bone marrow-derived mesenchymal stromal cells for the treatment of allograft rejection after renal transplantation: results of a phase I study. Stem Cells Transl Med. 2013;2(2):107-111. | 6 KTRs of living-related kidney graft donors | **Induction:** basiliximab  **Maintenance**: prednisone, calcineurin inhibitor (tacrolimus or cyclosporine), and mycophenolate mofetil.  **Treatment group**:Patients with subclinical rejection in the renal biopsy at 4 weeks or 6 months or an increase in IF/TA in the biopsy 6 months after transplantation receive two doses of 1–2 × 10^6^ cells/kg bw 7 days apart. | **Design:** A safety and feasibility study to evaluate safety and efficacy of autologous bone marrow bmMSCs to treat rejection and/or an increase in interstitial fibrosis/tubular atrophy (IF/TA).  **Results:** 6 patients received MSC infusions. Clinical and immune monitoring was performed up to 24 weeks after MSC. Infusions were well-tolerated, no treatment-related serious adverse events. In two recipients with allograft rejection, there was a resolution of tubulitis without IF/TA after MSCs. Additionally, three patients developed an opportunistic viral infection, and five of the six patients displayed a donor-specific downregulation of the peripheral blood mononuclear cell proliferation assay, not reported in patients without MSC treatment.  **Conclusions:**  Autologous BM MSC treatment in transplant recipients with subclinical rejection and IF/TA is clinically feasible and safe, and the findings are suggestive of systemic immunosuppression. |
| 1. Peng Y, Ke M, Xu L, et al. Donor-derived mesenchymal stem cells combined with low-dose tacrolimus prevent acute rejection after renal transplantation: a clinical pilot study. Transplantation. 2013 Jan 15;95(1):161-8. | 6 KTRs of living-related kidney graft donors | **Treatment group:**  donor-derived bmMSCs combined with a sparing dose of tacrolimus (50% of standard dose)  **Control group:**  standard dose of tacrolimus. | **Design:**Safety and efficacy of donor-derived bmMSCs combined with a sparing dose of tacrolimus (50% of standard dose) vs recipients treated with standard dose of tacrolimus.  **Results:**  None of the MSC recipients experienced immediate or long-term toxic side effects associated with MSC infusion. The tacrolimus dose (0.045±0.002 mg/kg) in the MSC group was significantly reduced compared with the control group (0.077±0.005 mg/kg). One acute rejection occurred only in the control group. All patients survived with stable renal function at month 12 and no chimerism was detectable at month 3. Patients in the MSC group showed significantly higher B-cell levels than the control group at month 3.  **Conclusions:**  Data suggest that the use of MSCs could provide potential benefits in renal transplantation by reducing the dosage of conventional immunosuppressive drug that is required to maintain long-term graft survival and function. |
| 1. Vanikar AV, Trivedi HL, Kumar A, et al. Co-infusion of donor adipose tissue-derived mesenchymal and hematopoietic stem cells helps safe minimization of immunosuppression in renal transplantation - single center experience. Ren Fail. 2014;36(9):1376-1384. | 95 KTRs of living-related kidney graft donors | **Preconditioning:**  lymphoid irradiation and anti-thyroglobulin.  **Treatment groups:**  group-1 received portal co-infusion of AD-MSC + HSC (n=95),  group-2 received portal infusion of HSC (n=95)  group-3 received no SCT (n=95). | **Design:**  Comparison of 3 protocols: preconditioning with co-infusion of donor adipose-derived mesenchymal stem cells (AD-MSC) + hematopoietic stem cells (HSC) vs only HSC vs no SCT in living donor LDRT under non-myeloablative conditioning.  **Results:**   SCT was safe. At 1 and 5 years post-transplant, patient survival was 100% and 94.7% in group-1, 100% and 95.7% in group-2, and 94.7% and 84% in group-3, death-censored graft survival was 100% and 94.6% in group-1, 100% and 91.3% in group-2, and 98.9% and 94.4% in group-3 with mean serum creatinine (mg/dL) of 1.38 and 1.39 in group-1, 1.48 and 1.51 in group-2, and 1.29 and 1.42 and in group-3. Rejection episodes and immunosuppression requirement were lesser in SCT groups versus controls with best results noted in group 1.  **Conclusions:**  Coinfusion of donor AD-MSC +HSC in portal circulation pre-transplant under non-myeloablative conditioning is safe and effective for immunosuppression minimization in living-related kidney transplant recipients |
| 1. Pan GH, Chen Z, Xu L, et al. Low-dose tacrolimus combined with donor-derived mesenchymal stem cells after renal transplantation: a prospective, non-randomized study. Oncotarget. 2016;7(11):12089-12101. | 16 KTRs of living-related kidney graft donors | **Induction:** Cytoxan (200 mg/day) and methylprednisolone (750, 500, and 250 mg/day) from days 0 to 3.  **Maintenance:** mycophenolate mofetil (1 g/day) and prednisone (initiated at 30 mg/day at day 4 after kidney transplantation and then tapered by 5 mg every week to the maintenance dose of 15 mg/day).  **Control group:**  standard dose of tacrolimus (0.07-0.08 mg/kg/day).  **Treatment group:**  low dose of tacrolimus (0.04-0.05 mg/kg/day) and two infusions of MSCs, the first 5×10^6^ cells delivered into the renal allograft artery at the time of kidney transplantation, the second infusion (2×10^6^ cells/kg) delivered after one month intravenously. | **Design**  Donor-derived, bm MSCs combined with a sparing dose of tacrolimus compared to controls on a standard dose of tacrolimus.  **Results**  All patients survived and had stable renal function at the 24 month follow-up. The combination of low-dose tacrolimus and MSCs was as effective as standard dose tacrolimus in maintaining graft survival at least 2 years after transplantation. In addition, both groups had similar urea, urine protein, urinary RBC, urinary WBC, 24-h urine protein, and creatinine clearance rates from 7 days to 24 months after transplantation. Furthermore, no differences in the proportion of lymphocytes, CD19, CD3, CD34, CD38, and natural killer cells were detected between the control and experimental groups. None of the MSC recipients experienced immediate or long-term toxicity from the treatment.  **Conclusions:**  This data suggest that the addition of MSCs permits the use of lower dosages of nephrotoxic calcineurin inhibitors following renal transplantation. |
| 1. Sun Q, Huang Z, Han F, et al. Allogeneic mesenchymal stem cells as induction therapy are safe and feasible in renal allografts: pilot results of a multicenter randomized controlled trial. J Transl Med. 2018;16(1):52. | 21 KTRs of living-related kidney graft donors | **Induction:** antithymocyte globulin (50 mg/day) and methylprednisolone (500 mg/day) were continuously administered intravenously during the first 3 postoperative days.  **Maintenance:** prednisone, mycophenolate mofetil (daily dose of 1.0–1.5 g) and calcineurin inhibitor. Tacrolimus or cyclosporine was started on days 2–4 to achieve the target trough levels in the peripheral blood 5–8 ng/ml for tacrolimus, and 130–150 ng/ml for cyclosporine.  **Control group**:  Standard induction therapy  **Treatment group:**  Standard induction therapy+2 × 10^6^/kg human umbilical-cord-derived MSCs (UC-MSCs) via the peripheral vein before renal transplantation, and 5 × 10^6^ cells via the renal artery during the surgical procedure. | **Design:**  Safety and efficacy of standard induction therapy+ human umbilical-cord-derived MSCs (UC-MSCs) vs standard induction therapy in living related KTRs.  **Results:**  Treatment with UC-MSCs achieved comparable graft and recipient survivals with non-MSC treatment (P = 0.97 and 0.15, respectively). No increase in postoperative complications, including DGF and acute rejection, were observed (incidence of DGF: 9.5% in the MSC group versus 33.3% in the non-MSC group, P = 0.13; Incidence of acute rejection: 14.3% versus 4.8%, P = 0.61). Equal postoperative estimated glomerular filtration rates were found between the two groups (P = 0.88). All patients tolerated the MSCs infusion without adverse clinical effects. Additionally, a multiprobe fluorescence in situ hybridization assay revealed that UC-MSCs administered via the renal artery were absent from the recipient's biopsy sample.  **Conclusions:**  UC-MSCs can be used as clinically feasible and safe induction therapy. Adequate timing and frequency of UC-MSCs administration may have a significant effect on graft and recipient outcomes. |
| 1. Erpicum P, Weekers L, Detry O, et al. Infusion of third-party mesenchymal stromal cells after kidney transplantation: a phase I-II, open-label, clinical study. Kidney Int. 2019;95(3):693-707. | 10 KTRs from deceased donors | **Induction and maintenace therapy:**  tacrolimus, mycophenolate mofetil, and corticosteroids associated with anti–interleukin-2 receptor antibodies on day 0 and day 4.  **Control group:**  10 KTRs who declined MSC infusion but accepted supplemental immunological follow-up and whole cohort of KTRs fulfilling the inclusion/exclusion criteria who were transplanted during the study period.  **Treatment group:**  third-party bone marrow MSCs (∼2 × 10^6^/kg) on day 3 ± 2 post-transplant | **Design:**  To evaluate efficacy and safety of third-party bmMSCs compared to concurrent controls without MSCs therapy.  **Results:**  No adverse effects were noted at MSC injection. One participant with a history of cardiac disease had a non-ST-elevation myocardial infarction approximately 3 hours after MSC infusion. Incidences of opportunistic infections and acute rejection were similar. At day 7 post-transplant, eGFR in MSC-treated recipients reached 48.6 ml/min/1.73m^2^, compared to 32.5 ml/min/1.73m^2^ in controls and 29.3 ml/min/1.73m^2^ in our overall cohort of KTRs. No difference in eGFR was found at 1 year. MSC-treated recipients showed increased frequencies of regulatory T cells at day 30, with no significant change in B cell frequencies compared to concurrent controls. Four MSC-treated participants developed antibodies against MSC or shared kidney-MSC HLA, with only 1 with MFI >1500.  **Conclusions:**  A single infusion of third-party MSC following kidney transplantation appears to be safe, with one cardiac event of unclear relationship to the intervention. MSC therapy is associated with increased regulatory T cell proportion and with improved early allograft function. Long-term effects, including potential immunization against MSC, remain to be studied. |
| 1. Dreyer GJ, et al. Human leukocyte antigen selected allogeneic mesenchymal stromal cell therapy in renal transplantation: The Neptune study, a phase I single-center study. Am J Transplant. 2020 Oct;20(10):2905-2915. | 10 KTRs of living kidney graft donors | **Induction and maintenance therapy:**  alemtuzumab (anti‐CD52) induction and prednisone, tacrolimus and everolimus as maintenance therapy.  **Treatment group:**  two doses of allogenic* bmMSCs 1.5 × 10^6^ /kg 6 months post Ktx, combined with a sparing dose of tacrolimus (tacrolimus was reduced to trough levels of 1.5‐3 ng/mL after the second MSC infusion) in combination with everolimus and prednisone.* selected MSCs were used with a matching strategy that prevented repeated mismatches | **Design:**  To evaluate safety and feasibility of allogeneic MSCs in KTRs using a matching strategy that prevented repeated mismatches.  **Results:**  No biopsy proven acute rejection or graft loss occurred and renal function remained stable. No major alterations in T and B cell populations or plasma cytokines were observed upon MSC infusion, although one patient produced DSAs against MSCs, formed before infusion.  **Conclusions:**  Administration of HLA selected allogeneic MSCs combined with low‐dose tacrolimus 6 months after transplantation is safe at least in the first year after renal transplantation. |
| 1. Reinders MEJ, et al. Autologous bone marrow-derived mesenchymal stromal cell therapy with early tacrolimus withdrawal: The randomized prospective, single-center, open-label TRITON study. Am J Transplant. 2021 Feb 9. | 57 KTRs of living kidney graft donors | **Induction and maintenance therapy:**  alemtuzumab 15 mg sc, at days 0 and 1; tacrolimus, everolimus, and low-dose prednisone as maintenance therapy.  **Treatment group:**  autologous bone marrow MSCs (∼2x1.5 × 10^6^ per/kg body weight iv) at weeks 6 and 7 post-transplant+increased steroid dose 15 mg/d,  early tacrolimus withdrawal at week 8 (n=29)  **Control group:**  standard dose tacrolimus (n=28) | **Design:**  To compare MSCs infused 6 and 7 weeks after renal transplantation and early tacrolimus withdrawal with a control tacrolimus group. Primary end point is quantitative evaluation of interstitial fibrosis in protocol biopsies at 4 and 24 weeks posttransplant. Secondary end points included acute rejection, graft loss, death, renal function, adverse events, and immunological responses.  **Results:**  Early tacrolimus withdrawal with MSC therapy was safe and feasible without increased rejection (1 acute rejection (MSC group), 7 subclinical rejections in week 24 (4 MSC; 3 controls)) and with preserved renal function.  Quantitative progression of fibrosis in protocol biopsies at 4 and 24 weeks posttransplant showed no benefit in the MSC group.  **Conclusion:**  Early tacrolimus withdrawal with MSC therapy is safe and feasible without increased rejection and with preserved renal function. |
| 1. Ban TH, Lee S, Kim HD, et al. Clinical Trial of Allogeneic Mesenchymal Stem Cell Therapy for Chronic Active Antibody-Mediated Rejection in Kidney Transplant Recipients Unresponsive to Rituximab and Intravenous Immunoglobulin. Stem Cells Int. 2021;2021:6672644. Published 2021 Feb 10. doi:10.1155/2021/6672644 | 2 KTRs from deceased donors | **Induction and maintenance therapy:**  Tacrolimus (trough level 3-8 ng/ml), mycophenolate mofetil, and corticosteroids associated with 2 corticosteroid iv doses of 500 mg (applied 2 months prior to MSCs therapy), followed by 30 mg daily until MSCs therapy.  **Treatment group:**  third party bone marrow MSCs (4 × 10^6^/kg) administered every other week iv. | **Design:**  To evaluate safety and efficacy of allogenic bmMSC in KTRs with chronic AMR unresponsive to rituximab and intravenous immunoglobulin.  **Results:**  No serious adverse events during the 6 months study period. Renal function was stable during MSC treatment but gradually decreased between the final MSC infusion and the study endpoint (patient 1: creatinine levels ranged from 3.01 mg/dL to 7.81 mg/dL, patient 2: 2.87 mg/dL to 3.91 mg/dL). In peripheral blood sample analysis between the start of treatment and 3 months after the final MSC infusion, there were similar trends for immunomodulatory markers.  **Conclusion:**  There were no serious adverse events for six months after allogeneic MSC treatment but also no obvious efficacy of MSC treatment was detected in patients with chronic AMR refractory to rituximab and intravenous immunoglobulin, |
| 1. Wei Y, Chen X, Zhang H, et al. Efficacy and Safety of Bone Marrow-Derived Mesenchymal Stem Cells for Chronic Antibody-Mediated Rejection After Kidney Transplantation- A Single-Arm, Two-Dosing-Regimen, Phase I/II Study. *Front Immunol*. 2021;12:662441. Published 2021 Jun 25. doi:10.3389/fimmu.2021.662441. | 23 KTRs (kidney donor type not specified) | **Maintenance therapy:**  calcineurin inhibitors, mycophenolate with or without glucocorticoids.  **Treatment group:**  Patients were treated with bmMSCs as the initial regimen or second-line regimen after previous failed AMR treatment, including plasmapheresis, intravenous immunoglobulin, rituximab, bortezomib and methylprednisolone.  Allogenic third party MSCs 1.0×10^6^ cells/kg per month for four consecutive months (Regimen 1; n=8) or 1.0×10^6^ cells/kg per week for four consecutive weeks (Regimen 2;n=15).  **Control group:**  Contemporaneous cAMR patients who did not receive bmMSCs were retrospectively analyzed (n =30). | **Design:**  To investigate the efficacy and safety of bmMSCs on chronic AMR in the kidney allograft.  **Results:**  The median delta eGFR of the total bmMSCs treated patients was -4.3 ml/min/1.73m^2^ vs the median delta eGFR of the control group -12.7 ml/min/1.73 m^2^2 years after bmMSCs treatment (P=0.0233). The median delta maximum donor-specific antibody was significantly greater than that of the bmMSCs treated group (P=0.0342). The incidence of hepatic enzyme elevation, BK polyomaviruses (BKV) infection, cytomegalovirus (CMV) infection was 17.4%, 17.4%, 8.7%, respectively. There was no side effects. Flow cytometry analysis showed a significant decreasing trend of CD27^-^IgD^-^ double negative B cells subsets and trend towards the increase of CD3^+^CD4^+^PD-1^+^/lymphocyte population after MSCs therapy. Multiplex analysis found TNF-α, CXCL10, CCL4, CCL11 and RANTES decreased after MSCs treatment.  **Conclusion**  Kidney allograft recipients with cAMR are tolerable to bmMSCs which can delay the deterioration of allograft function, probably by decreasing DSA level and reducing DSA-induced injury. The underlying mechanism may involve immunomodulatory effect of MSCs on peripheral B and T cells subsets. |

**Study inclusion and exclusion criteria**

The inclusion and exclusion criteria were as follows: adult recipients (>18 years) of a kidney graft with histologicaly proven chronic active AMR and an estimated glomerular filtration rate (eGFR) >20 ml/min/1.73 m^2^, written informed consent, compliant with local regulations. Exclusion criteria included recipients of multiple organs, pregnancy, history of malignant disease in the last 5 years, active autoimmune disease, active infection including hepatitis B, hepatitis C, HIV, or tuberculosis, evidence of congestive cardiac failure and/or acute coronary syndrome in past 6 months, evidence of liver disease, and inadequate compliance to treatment.

**Mesenchymal stem cell preparation-details on characteristics and culture protocol**

Cell therapy MSCs products were prepared at two different sites, following GMP guidelines. Slightly different protocols for MSCs expansion were used; however, release criteria were the same for both facilities. Briefly, patients’ bone marrow was harvested after signing the informed consent and prior to receiving standard of care therapy for chronic AMR. Bone marrow harvest was performed according to institutional protocol under general anesthesia (Patient#1 and Patient#3) and local anesthesia (Patient#2) by experienced hematology team and maximum 400 ml of bone marrow was harvested per patient. Bone marrow (296 ml for patient#1, 23,1 ml Patient#2 and 217 ml for patient#3) was tested for sterility and mycoplasma. Cell culture media were MEMα + 5 % platelet lysate (Patient#1 and Patient#3) and DMEM/F-12 + 10 % human serum + 4 ng/ml bFGF (Peprotech, USA) (Patient#2); (Gibco, USA and Blood Transfusion Centre of Slovenia, Slovenia). Gentamicin 50 µg / ml (Garamycin, Krka, Slovenia) and heparin 2 i.u. / ml (Heparin, B. Braun Mesulgen, Germany) were added to culture media. For Patient#1 and Patient#3 gentamycin was employed throughout the cultivation process, while for the patient#2 only in the primary culture. For subculturing, Trypsin-EDTA (Patient#1 and Patient#3) or TrypLE™ Select CTS™ (Patient#2) were used (both Gibco, USA). For cryopreservation 10% DMSO (CryoSure-DMSO, WAK-Chemie Medical, Germany) was combined with human albumin (Albunorm, Octapharma, Great Britain) or human serum (Blood Transfusion Centre of Slovenia, Slovenia) for Patient#1/Patient#3 and Patient#2, respectively. For the treatment fresh cells from passages 1, 2 and 3 (Patient#1/Patient#3) and passage 4 and 5 (Patient#2) were used. Final formulation (individual dose) contained 1 x 10^6^ cells / kg of patient body weight at a concentration of 1 x 10^6^ cells /ml in 0,5 % human albumin (Patient#1) and in 10% human albumin (Patient#2) (Albunorm, Octapharma, Great Britain).

Quality control of the products was conducted by flow cytometry phenotypic characterization, sterility, mycoplasma, and bacterial endotoxins testing. Details on MSCs preparation characteristics, culture protocol, viability and phenotypic characteristics of stem cell therapy administered to patient#1, patient#2 and patient#3 are presented in **Supplementary Table 2A. In Supplementary Table 2B** viability and phenotypic characteristics of stem cell therapy administered to patient#1 and patient#2 are presented. Data for patient#3 are reported elsewhere (doi: 10.3389/fmed.2021.708744). Criteria for MSC batch release were the following: expression of CD105, CD73, and CD90 > 70 %, CD45< 10 %; negative for mycoplasma, Gram-positive, and Gram-negative bacteria, and fungi; endotoxin below 0,5 EU / ml; viability > 70 %.

**Supplementary Table 2A: The comparison of cultivations for patients.**

|  | **Patient #1** | **Patient #2** | **Patient #3** |
| --- | --- | --- | --- |
| Quantity of bone marrow used | 296 ml | 23,1 ml | 217 ml |
| Cell culture media | MEMα | DMEM | MEMα |
| bFGF | Not utilized | utilized | not utilized |
| gentamycin | Throughout cell culture | Primary culture only | Throughout cell culture |
| Trypsinization agent | trypsin | TrypLE™ Select CTS™ | trypsin |
| Freezing solution | DMSO + human albumin | DMSO + human serum | DMSO + human albumin |
| Cells used for infusion | 3P, ,1P, 2P (for respective infusions  I, II, III) | 4P (I) and 5P (II) | 2P (I, II, III) |
| General impression | No deviations | Impaired culture growth compared to healthy donor cells | Altered cell morphology (higher percentage of flattened cells in early culture) |

For patient#1 there were no deviations from culturing protocol, yields were as expected. We used gentamicin routinely as a preventive measure against microbiological outgrowth. The first preparation contained cells from the third passage of the cell culture, second and third MSC infusions were prepared from thawed and cultured cells from the first and second passage.

For patient#2 there were also no deviations from culturing protocol, yields were as expected. However, the cells generally have not performed well compared to our experience with healthy donor cells – the growth was slower, and they detached easily from the surface, which is also seen in other immune diseases (unpublished own data). We used gentamicin solely during primary culture as a preventive measure against microbiological contaminants from donor bone marrow, after primary culture cells were cultivated without gentamycin.

Expressed values of CD73, CD 90, CD105 and CD45 were all in acceptable ranges and thus complied to release criteria (Supplementary Table 2B). Cells expressed low levels of HLA-DR (patient#1) and CD34 (patient#2). Platelet lysate and human sera tested negative for routinely screened markers in blood donors (HBsAg, anti- HCV, anti- HIV I/II and HIV Ag, anti-TP antibodies and NAT for HBV, HCV, HIV) and were negative in sterility testing (BAC TEC).

**Supplementary Table 2B: Autologous MSCs cell infusion characteristics in patient #1 and in patient #2**

| **Patient #1** | |  | Phenotype | | | | |  |
| --- | --- | --- | --- | --- | --- | --- | --- | --- |
| Application |  | Viability | CD 73 | CD 90 | CD105 | CD45 | HLA-DR | Sterility/Mycoplasma/Bacterial endotoxin |
| 1st | | 98.9 | 100.0 | 99.7 | 81.2 | 0.2 | 3.6 | Comply |
| 2nd | | 97.9 | 99.6 | 98.8 | 96.5 | 0.7 | 0.5 | Comply |
| 3rd | | 99.7 | 99.9 | 99.3 | 92.2 | 2.0 | 1.9 | Comply |
| **Patient #2** | |  | Phenotype | | | | |  |
| Application | | Viability | CD 73 | CD 90 | CD105 | CD45 | CD34 | Sterility/Mycoplasma/Bacterial endotoxin |
| 1st | | 92.8 | 91.2 | 85.4 | 95.7 | 0.1 | 0.1 | Comply |
| 2nd | | 92.8 | / | / | / | / | / | Comply |

**RNA isolation and miRNA quantification**

All the reagents were from Qiagen (Hilden, Germany) except where otherwise indicated. Total RNA isolation was performed using 200 µl of serum and miRNeasy serum/plasma advanced kit (Qiagen, Hilden, Germany) according to the manufacturer’s protocol. RNA was eluted in 20 µl RNase-free water. The successful isolation procedure was confirmed by adding spike-ins UniSp2, UniSp4, UniSp5 (RNA Spike-in Kit, for RT; Qiagen, Hilden, Germany) and subsequent quantification of these spike-ins.

Quantitative real-time polymerase chain reaction (qPCR) was carried out using Rotor Gene Q. miRNAs (*hsa-miR-29c, hsa-miR-126, hsa-miR-146a, hsa-miR-150, hsa-miR-155, hsa-miR-223*) were analysed relatively to the reference genes, *hsa-miR-103a-3p*, *hsa-miR-191* and *hsa-miR-423.* The possibility of homeolysis was excluded by quantifying *hsa-miR-23a* and *hsa-miR-451a*. The succesfull RT was estimated analysing expression of UniSp6. All qPCR reactions were caried out using the miRCURY LNA miRNA PCR assay according to the manufacturer’s instruction.

Prior to qPCR, reverse transcription was performed using a miRCURY LNA RT Kit in a 10 μl reaction master mix, containing 2 µl of total RNA and UniSp6 spike-in according to the manufacturer’s instructions. The resulting RT was diluted 20-fold and 3 μl was used in 10 μl reaction master mix (miRCURY LNA SYBR Green PCR Kit), according to the manufacturer’s instructions. All the qPCR reactions were performed in duplicate.

Also prior to qPCR, RNA samples were pooled, followed by RT and qPCR as described above. Efficiency was tested for each analyzed miRNA using 10-fold dilutions and qPCR was performed in triplicate.

For all analyses, the signal was collected at the endpoint of every cycle. Following amplification, melting curve analysis of PCR products was performed to verify the specificity and identity. Melting curves were acquired on the SYBR channel using a ramping rate of 0.7°C/60 s for 60–95°C.

**Immunological monitoring of HLA, anti-ETAR and anti-ATR1 antibodies**

For HLA antibody analysis, serum samples were screened using Luminex screen assay (Lifecodes, Immucor) and analyzed with a Luminex 200 reader. When positive, a single antigen bead (SAB) assay (Lifecodes, Immucor) was performed as standard-of-care. Assignment of positivity was assessed according to the manufacturer's instructions. Quantitative determination of the anti-AT1R and anti-ETAR antibodies were measured with a sandwich ELISA (One Lambda, Canoga Park, CA, USA).

**Lymphocyte phenotyping and function**

To analyze lymphocyte populations, peripheral blood was collected from patients at the time of the first kidney graft biopsy and later 1, 2, 3, 6, 9 and 12 months after MSC transplantation. All samples were run at a single site (Institute of Microbiology and Immunology, Medical Faculty, University of Ljubljana, Slovenia). Proportions of CD3^+^, CD4^+^ T (CD3^+^CD4^+^), CD8^+^ T (CD3^+^CD8^+^), CD4^+^/CD8^+^ ratio, B (CD19^+^); activated T (CD3^+^HLA DR^+^), activated CD4^+^T (CD25^+^CD4^+^), NK (CD16^+^CD56^+^), naive T (CD4+CD45RA^+^), memory T (CD4^+^CD45RA^-^), RTE (CD4^+^CD45RA^+^CD31^+^), TCR αβ DNT (CD3^+^CD4^-^CD8^-^ TCRαβ^+^), TCR γδ DNT (CD3^+^CD4^-^CD8^-^ TCRγδ^+^), αβ (CD3^+^ TCRαβ^+^), γδ (CD3^+^ TCRγδ^+^), Th1 (cIFNγ^+^CD4^+^), Th2 (cIL4^+^CD4^+^), Th17 (cIL17^+^CD4^+^), CD4^+^CD25^++^ (ie. CD4^+^CD25^high^ cells, which may contain a fraction of Tregs) cell subsets were determined by flow cytometry. Peripheral blood cytokine analysis was not part of the study protocol, but we started performing it after patient 3 suffered severe side effects with the aim of elucidating only these. Therefore we decided to define cytokines from a certain time point also in the remaining 2 patients. We determined the concentration of the following cytokines: TNF- α, IFN-γ , IL-1 β, IL-10, IL-12, IL- 6, IL-8.

**Supplementary Table 3. Chronological report of immune monitoring studies in patient #1.** Legend: CD3^+^-T lymphocytes, CD19^+^-B lymphocytes, CD4^+^-T helper lymphocytes, CD8^+^-cytotoxic T lymphocytes, CD3^+^HLADR^+^-activated T lymphocytes, CD16^+^CD56^+^-natural killer cells, CD45 RA-naive T lymphocytes, CD4^+^CD45RA^-^ memory T lymphocytes, CD45RA^+^ CD31^+^- CD4RTE T lymphocytes, Treg- CD4^+^CD25^++^ T cells ((ie. CD4^+^CD25^high^ cells, which may contain a fraction of Tregs).), CD4^-^CD8^-^ TCRγδ ^+^-T cells with TCRγδ, CD4^-^CD8^-^ TCRαβ^+^-T cells with TCRαβ.1st KTx biopsy-the time of 1st kidney transplant biopsy.

| **Date of assay** | **CD3+ [%]** | **CD3+ conc.** | **CD19+ [%]** | **CD19+ conc.** | **CD4+ [%]** | **CD4+ conc.** | **CD8+ [%]** | **CD8+ conc.** | **CD3+HLA-DR+ [%]** | **CD3+HLA-DR+ conc.** | **CD4/CD8 ratio** | **CD16+CD56+ [%]** | **CD16+CD56+ conc.** | **CD4+CD45RA+ [%]** | **CD4+CD45RA+ conc.** | **CD4+CD45RO+ [%]** | **CD4+CD45RO+ conc.** | **RTE [%]** | **RTE conc.** | **CD4-CD8- TCRαβ+ [%]** | **CD4-CD8- TCRγδ+ [%]** | **CD3+ TCRαβ+ [%]** | **CD3+ TCRγδ+ [%]** | **Th1 [%]** | **Th2 [%]** | **Th17 [%]** | **CD4+CD25+ [%]** | **Treg [%]** |
| --- | --- | --- | --- | --- | --- | --- | --- | --- | --- | --- | --- | --- | --- | --- | --- | --- | --- | --- | --- | --- | --- | --- | --- | --- | --- | --- | --- | --- |
| 1st KTx biopsy | 71 | 2.046 | 9 | 0.265 | 41 | 1.192 | 27 | 0.784 | 24 | 0.491 | 1.52 | 19 | 0.562 |  |  |  |  |  |  |  |  |  |  | 24 | 1 | 1 | 96 | 38 |
| 1m | 73 | 1.793 | 15 | 0.378 | 51 | 1.235 | 21 | 0.521 | 14 | 0.251 | 2.37 | 11 | 0.264 |  |  |  |  |  |  |  |  |  |  | 11 | 0.4 | 16 | 48.1 | 17.7 |
| 3m | 81 | 2.272 | 12 | 0.329 | 56 | 1.556 | 24 | 0.665 | 21 | 0.477 | 2.34 | 7 | 0.19 | 32 | 0.498 | 68 | 1.058 | 11 | 0.171 | 1 | 2 | 98 | 2 | 17 | 0.4 | 1.4 | 11 | 1.5 |
| 6m | 81 | 3.289 | 10 | 0.413 | 47 | 1.915 | 32 | 1.314 | 37 | 1.217 | 1.46 | 9 | 0.356 | 34 | 0.651 | 66 | 1.264 | 11 | 0.211 | 1 | 2 | 98 | 2 | 16 | 0.2 | 1 | 32.6 | 1.1 |
| 9m | 82 | 3.758 | 8 | 0.386 | 48 | 2.196 | 32 | 1.457 | 33 | 1.240 | 1.51 | 9 | 0.389 | 31 | 0.681 | 69 | 1.515 | 8 | 0.176 | 2 | 3 | 97 | 3.2 |  |  |  | 27 | 2 |
| 12m | 80 | 3.065 | 8 | 0.325 | 45 | 1.712 | 33 | 1.266 | 26 | 0.797 | 1.35 | 11 | 0.41 | 32 | 0.548 | 68 | 1.164 | 8 | 0.137 | 1 | 3 | 96 | 4 | 22 | 1 | 1 | 28 | 1 |

**Supplementary Table 4. Chronological report of immune monitoring studies in patient #2.** Legend: CD3^+^-T lymphocytes, CD19^+^-B lymphocytes, CD4^+^-T helper lymphocytes, CD8^+^-cytotoxic T lymphocytes, CD3^+^HLADR^+^-activated T lymphocytes, CD16^+^CD56^+^-natural killer cells, CD45 RA-naive T lymphocytes, CD4^+^CD45RA^-^ memory T lymphocytes, CD45RA^+^ CD31^+^- CD4RTE T lymphocytes, Treg- CD4^+^CD25^++^ T cells ((ie. CD4^+^CD25^high^ cells, which may contain a fraction of Tregs).), CD4^-^CD8^-^ TCRγδ ^+^-T cells with TCRγδ, CD4^-^CD8^-^ TCRαβ^+^-T cells with TCRαβ. 1st KTx biopsy-the time of 1st kidney transplant biopsy.

| **Date of assay** | **CD3+ [%]** | **CD3+ conc.** | **CD19+ [%]** | **CD19+ conc.** | **CD4+ [%]** | **CD4+ conc.** | **CD8+ [%]** | **CD8+ conc.** | **CD3+HLA-DR+ [%]** | **CD3+HLA-DR+ conc.** | **CD4/CD8 ratio** | **CD16+CD56+ [%]** | **CD16+CD56+ conc.** | **CD4+CD45RA+ [%]** | **CD4+CD45RA+ conc.** | **CD4+CD45RO+ [%]** | **CD4+CD45RO+ conc.** | **RTE [%]** | **RTE conc.** | **CD4-CD8- TCRαβ+ [%]** | **CD4-CD8- TCRγδ+ [%]** | **CD3+ TCRαβ+ [%]** | **CD3+ TCRγδ+ [%]** | **Th1 [%]** | **Th2 [%]** | **Th17 [%]** | **CD4+CD25+ [%]** | **Treg [%]** |
| --- | --- | --- | --- | --- | --- | --- | --- | --- | --- | --- | --- | --- | --- | --- | --- | --- | --- | --- | --- | --- | --- | --- | --- | --- | --- | --- | --- | --- |
| 1st KTx biopsy | 80 | 1.295 | 10 | 0.166 | 30 | 0.483 | 45 | 0.733 | 51 | 0.660 | 0.66 | 9 | 0.146 |  |  |  |  |  |  |  |  |  |  |  |  |  |  |  |
| 1m | 74 | 1.402 | 16 | 0.306 | 30 | 0.562 | 39 | 0.743 | 34 | 0.477 | 0.76 | 10 | 0.182 |  |  |  |  |  |  |  |  |  |  | 24.7 | 0.4 | 0.2 | 18.8 | 3.1 |
| 2m | 75 | 1.409 | 14 | 0.268 | 32 | 0.612 | 38 | 0.719 | 41 | 0.578 | 0.85 | 11 | 0.202 | 18 | 0.110 | 82 | 0.502 | 5 | 0.031 | 2 | 5 | 94 | 6 | 35 | 2 | 1 | 24 | 3.4 |
| 3m | 76 | 1.355 | 12 | 0.22 | 36 | 0.643 | 35 | 0.619 | 40 | 0.542 | 1.04 | 11 | 0.205 | 16 | 0.103 | 84 | 0.540 | 5 | 0.032 | 3 | 5 | 94 | 6 | 27 | 1 | 0.4 | 19 | 3 |
| 6m | 77 | 1.202 | 12 | 0.185 | 33 | 0.514 | 39 | 0.604 | 47 | 0.565 | 0.85 | 11 | 0.170 |  |  |  |  |  |  |  |  |  |  |  |  |  |  |  |
| 9m | 72 | 1.386 | 19 | 0.364 | 31 | 0.592 | 36 | 0.703 | 33 | 0.457 | 0.84 | 9 | 0.179 | 17 | 0.101 | 83 | 0.491 | 5 | 0.030 | 3 | 6 | 94 | 6 | 26.2 | 0.6 | 0.3 | 18 | 1 |
| 12m | 73 | 1.176 | 17 | 0.270 | 32 | 0.513 | 37 | 0.593 | 36 | 0.423 | 0.86 | 10 | 0.160 | 17 | 0.087 | 83 | 0.426 | 5 | 0.026 | 2 | 6 | 93 | 7 | 39.8 | 1.4 | 0.6 | 19 | 2 |

**Supplementary Table 5. Kidney allograft ultrasound Doppler imaging results prior to and 12 months post MSCs therapy in patient #1 and patient #2**

| **Prior to MSCs therapy** | **12 months post MSCs therapy** |
| --- | --- |
| **Patient #1** |  |
| **I. Morphology** |  |
| Position: L iliac  Longitudinal diameter. (cm): 13.2  Transverse diameter. (cm): 5.9x6.0  Surface: flat  Parenchyma: more echogenic  Parenchyma thickness (mm): 16.5  Description: no morphological specifities  Hollow system: slightly wider, calyxes transversely measuring up to 10 mm, pyelone up to 4.5 mm.  Stones / Cysts / Liquid collections: not visible | Position: L iliac  Longitudinal diameter. (cm): 12.2  Transverse diameter. (cm): 5.5x5.6  Surface: flat  Parenchyma: more echogenic  Parenchyma thickness (mm): 15.0  Description: no morphological specifities  Hollow system: normal.  Stones / Cysts / Liquid collections: not visible |
| **II. Doppler** |  |
| Perfusion: good  RI: 0.68/0.68  Acceleration time (ms): 47/40  Distance from distal vessel to capsule (mm): 3.0  Acceleration index (m/s2): 3.2/3.4  Blood flow rate at the end of diastole (cm/s): 9.0/8.6 | Perfusion: good  RI: 0.68/0.68  Acceleration time (ms): 56/53  Distance from distal vessel to capsule (mm): 2.0  Acceleration index (m/s2): 2.6/2.0  Blood flow rate at the end of diastole (cm/s): 9.6/7.3 |
| **III. Doppler of renal artery/vein:** Velocities post anastomosis are measured up to 0.85/0.21 m/s (measured without angular correction). A signal in the renal vein is present. | Velocities post anastomosis are measured up to 1.7/0.55 m/s (measured without angular correction). A signal in the renal vein is present |
| **Patient #2** |  |
| Position: L iliac  Longitudinal diameter. (cm): 11.7  Transverse diameter. (cm): 5.6.5.5  Surface: flat  Parenchyma: more echogenic  Parenchyma thickness (mm): 12-15  Description: no morphological specifities  Hollow system: slightly wider, calyxes transversely measuring up to 12 mm, pyelone up to 5 mm.  Stones / Cysts / Liquid collections: not visible | Position: L iliac  Longitudinal diameter. (cm): 11.8  Transverse diameter. (cm): 5.0x5.5  Surface: flat  Parenchyma: more echogenic  Parenchyma thickness (mm): 11-14  Description: no morphological specifitiesu  Hollow system: slightly wider calyxes of upper group.  Stones / Cysts / Liquid collections: not visible |
| **II. Doppler** |  |
| Perfusion: lower cortical perfusion density  RI: 0.74/0.78  Acceleration time (ms): 40/40  Distance from distal vessel to capsule (mm): 2.0  Acceleration index (m/s2): 5.6/6.2  Blood flow rate at the end of diastole (cm/s): 10.6/10.1 | Perfusion: good  RI: 0.77/0.80  Acceleration time (ms): 31/58  Distance from distal vessel to capsule (mm): 3.0  Acceleration index (m/s2): 6.8/4.1  Blood flow rate at the end of diastole (cm/s): 7.7/6.9 |
| **III. Doppler of renal artery/vein** |  |
| Velocities post anastomosis are measured up to 1.6/0.1 m/s (measured with angular correction). A signal in the renal vein is present. | Velocities post anastomosis are measured up to 1.4/0.27 m/s (measured with angular correction). A signal in the renal vein is present. |

**Supplementary Figure 1. Timely expression of preselected serum miRNAs in patient #1 and patient #2.** Control experiments for successful isolation and reverse transcription, to exclude the hemolysis and expression of reference genes are summarized in Supplementary Figure 2 (A-F). Legend: time point 1, time point before treatment; time points 2-5, approx. 3-months’ period starting from months 1 and finishing 1 year after treatment.

**
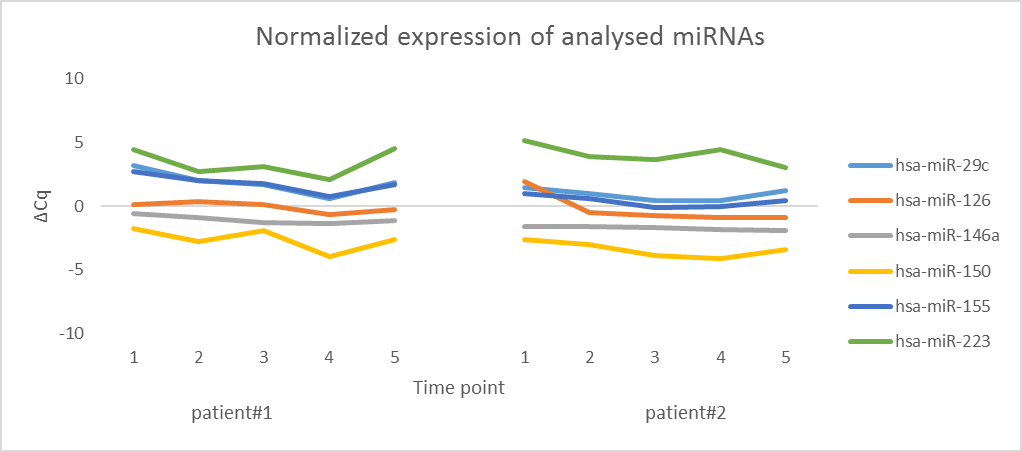
**

**Supplementary Figure 2**. **Control of isolation. reverse transcription. hemolysis and qPCR for expression analysis of miRNAs.** A, Expression of spike-ins (UniSp2, UniSp4, UniSp5) added as controls of isolation of RNA from serum; B, expression of spike-in (UniSp6) added as control of reverse transcription reaction; C, expression of miRNAs *hsa-miR-23a* and *hsa-miiR-451a;* D, difference in quantitation cycles of *hsa-miR-23a* and *hsa-miiR-451a* as control of hemolysis of the sample; E, expression of reference genes for miRNA normalization; F, expression of miRNAs before normalization. Legend: time point 1, time point before treatment; time points 2-5, approx. 3-months’ period starting from months 1 and finishing 1 year after treatment.


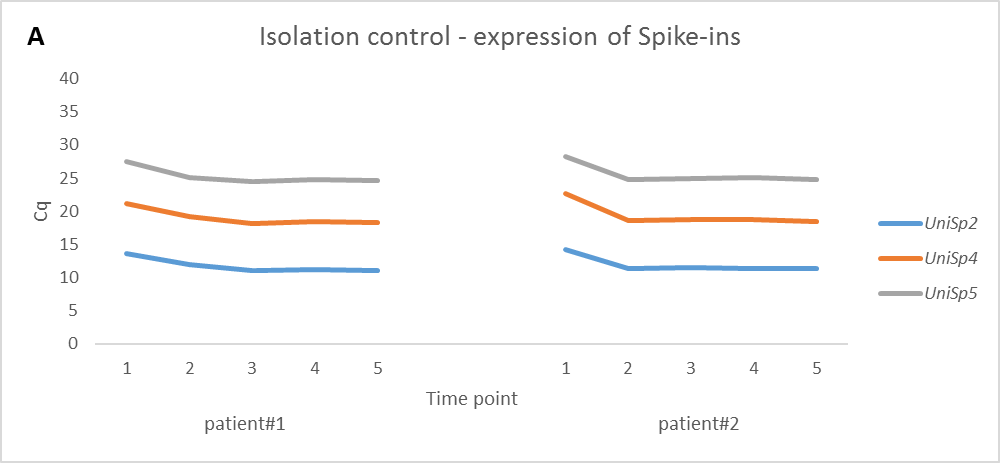


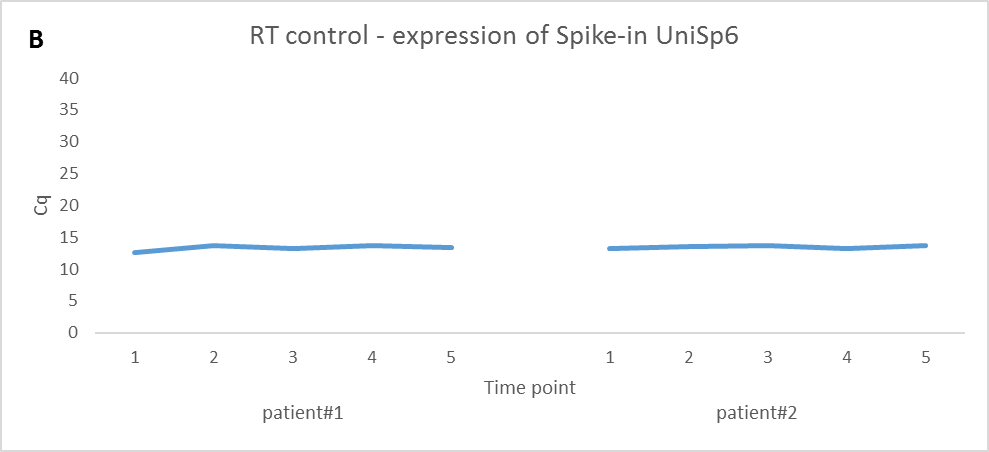


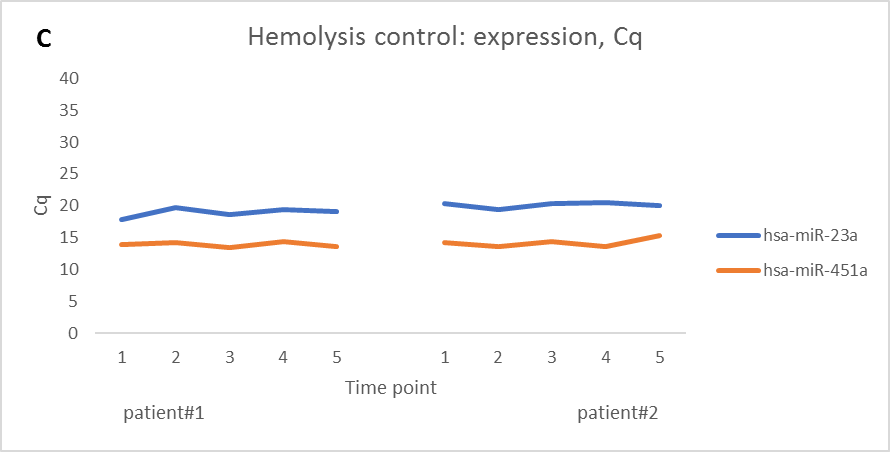


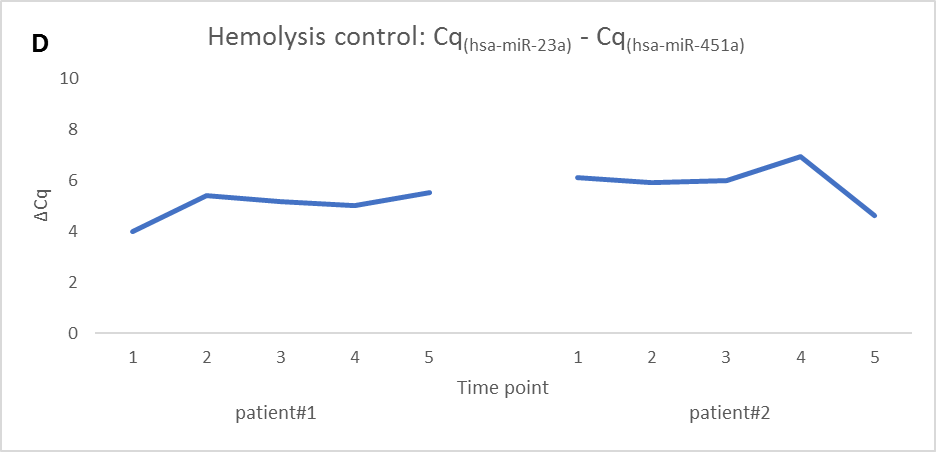


**
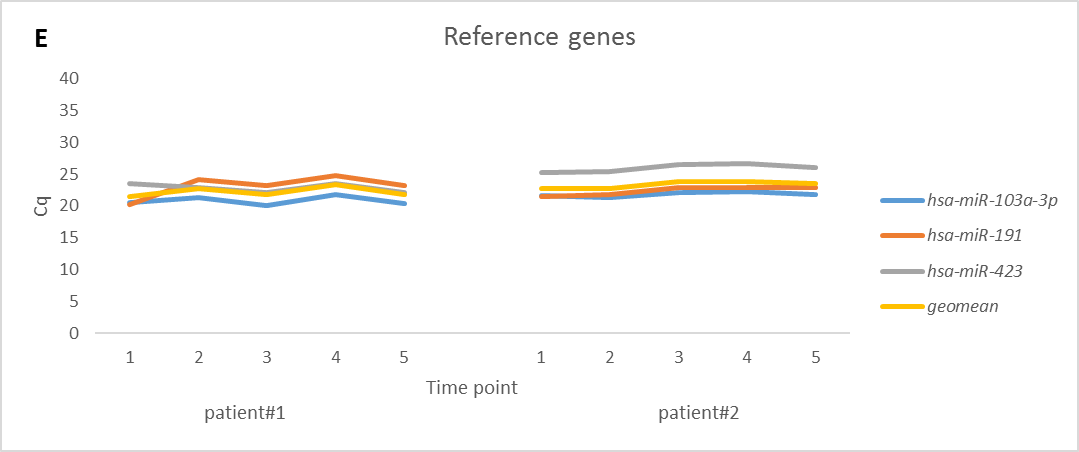
**

**
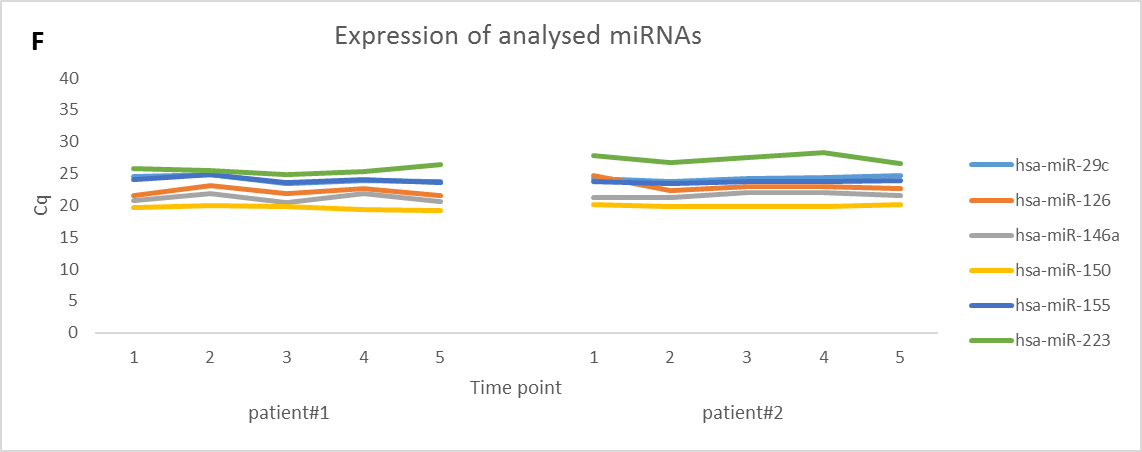
**
